# Self-Assembly of Block Copolymers in Thin Films Swollen-Rich in Solvent Vapors

**DOI:** 10.3390/polym15081900

**Published:** 2023-04-15

**Authors:** Iulia Babutan, Otto Todor-Boer, Leonard Ionut Atanase, Adriana Vulpoi, Ioan Botiz

**Affiliations:** 1 Interdisciplinary Research Institute on Bio-Nano-Sciences, Babeș-Bolyai University, 400271 Cluj-Napoca, Romania; iulia.babutan@ubbcluj.ro (I.B.); adriana.vulpoi@ubbcluj.ro (A.V.); 2 Faculty of Physics, Babeș-Bolyai University, 400084 Cluj-Napoca, Romania; 3 INCDO-INOE 2000, Research Institute for Analytical Instrumentation, 400293 Cluj-Napoca, Romania; otto.todor@icia.ro; 4 Department of Biomaterials, Faculty of Medical Dentistry, “Apollonia” University of Iasi, 700511 Iasi, Romania; leonard.atanase@univapollonia.ro; 5 Academy of Romanian Scientists, 050045 Bucharest, Romania

**Keywords:** block copolymers, thin films, solvent vapor annealing, self-assembly, atomic force microscopy

## Abstract

In this study we have employed a polymer processing method based on solvent vapor annealing in order to condense relatively large amounts of solvent vapors onto thin films of block copolymers and thus to promote their self-assembly into ordered nanostructures. As revealed by the atomic force microscopy, a periodic lamellar morphology of poly(2-vinylpyridine)-*b*-polybutadiene and an ordered morphology comprised of hexagonally-packed structures made of poly(2-vinylpyridine)-*b*-poly(cyclohexyl methacrylate) were both successfully generated on solid substrates for the first time.

## 1. Introduction

Polymer molecules are soft materials of relatively high molecular mass composed of multiple chemically bonded repeating units knows as monomers [1]. Depending on the chemical structure of monomers, the resulting polymer molecules can exhibit a variety of properties ranging from simple plastic [2,3,4] to biological [5,6], piezoelectric [7,8], thermoelectric [9,10], magnetic [11,12], or optoelectronic [13,14,15,16] functions, just to name a few. Therefore, it is not surprising at all that polymers are being used in an increasing number of applications across many multidisciplinary fields [17,18,19,20,21,22,23,24]. Nonetheless, to maximize the utility of polymers and to precisely tune and control their functions and properties, one needs to control the resulting microstructure at multiple length scales, stretching from the micrometer scale down to the nanometer scale, as there is a clear correlation between the structural arrangements of polymer molecules and their various properties [6,16,25,26,27].

Consequently, there is a clear need for the development of new polymer processing techniques in order to precisely manipulate these materials in bulk, in solutions and especially on surfaces. Many of these techniques may rely on ordering processes such as polymer self-assembly [28,29,30,31,32,33,34] or crystallization [33,35,36,37,38]. In particular, the self-assembly process becomes of paramount importance when block copolymers (BCPs) are used to generate hierarchically ordered micro- and nanostructures for various technological applications [39,40,41,42], including the BCP lithography [23,39,40]. Common polymer/BCP processing methods known to manipulate and influence the self-assembly process are based on a wide range of physical and physicochemical approaches [43,44,45] and may include the utilization of space confinements [42,46,47,48,49] and solvent vapor annealing [50,51,52,53]. 

**Figure 1:**
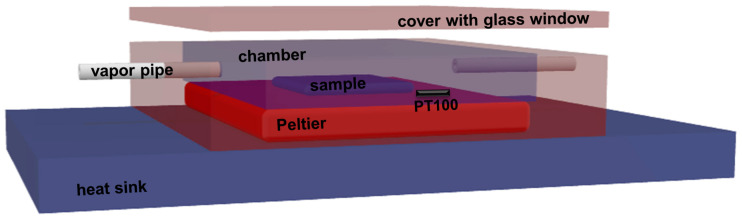
Schematics depicting the homemade sample processing chamber used to condense relatively large amounts of solvent vapors onto thin films of BCPs and thus swell-rich these polymer films. In this experimental setup, a Peltier module provides precise heating/cooling of the sample, which is driven by a temperature controller equipped with a PT100 temperature sensor. Moreover, while the heat sink was coupled to a fan (not depicted in the schematics), the solvent pipe was connected to a nitrogen-based “bubbling” system (also not portrayed in this figure) that could pump precise amounts of solvent vapors from a solvent container into the sample chamber through a solvent vapor pipe. Note that the top chamber cover has a glass window that allows real-time and direct-space observations to be made by using an optical microscope. The dimensions utilized in this scheme are not drawn at the scale.

In this study, in order to favor the self-assembly of three different BCP systems and to reveal new ordered morphologies in thin films, the corresponding BCP thin films were swollen-rich using a recently validated processing method based on the solvent vapor annealing in a quasi-confined environment (C-SVA) [54]. The homemade equipment consisted of a sample chamber of reduced depth that acted as a quasi-confinement for the solvent and polymer molecules. Thin BCP films were placed inside the chamber and swollen through the introduction of precise amounts of solvent vapors using a nitrogen-based solvent “bubbling” system. Connected to a state-of-the-art temperature controller, the bottom of the chamber could maintain the film temperature constant over time, with a precision of 0.01 °C. Moreover, this setup, schematically presented in Figure 1, allowed the rate of temperature change to be finely tuned, eliminating the unwanted variations in the sample temperature that frequently occur when approaching a certain temperature setpoint. Consequently, the undesirable fluctuations in the film thickness around the temperature setpoint could be annihilated when BCP films were undergoing rich-swelling and became quasi 2D “solutions”. Compared to the previous setups, when either a centimeter-deep two-compartment solvent-sample chamber was employed along with a less precise control over the film temperature (in this case, weak fluctuations in film thickness, appearing when approaching a certain swollen state corresponding to a fixed temperature setpoint, were most often unavoidable) [55], or when a less confined single-component sample chamber was used [36,56], the here-presented setup allowed us to eliminate these disadvantages. Thus, the gradients of solvent vapors that often lead, under rich-swelling conditions, to post-processing film inhomogeneities (i.e., to films characterized by unequal thickness over their surfaces), could be mostly avoided.

## 2. Materials and Methods

**Figure 2:**
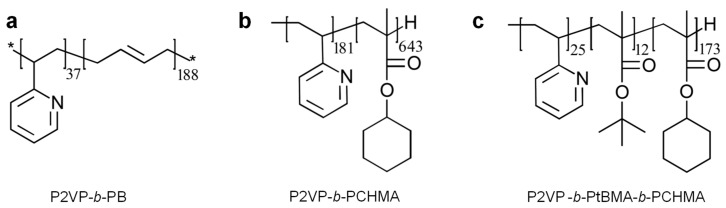
The chemical structures of P2VP_37_-*b*-PB_188_ (**a**), P2VP_181_-*b*-PCHMA_643_ (**b**) and P2VP_25_-*b*-P*t*BMA_12_-*b*-PCHMA_173_ (**c**) block copolymers studied in this work.

Poly(2-vinylpyridine)-*b*-polybutadiene (P2VP_37_-*b*-PB_188_), poly(2-vinylpyridine)-*b*-poly(cyclohexyl methacrylate) (P2VP_181_-*b*-PCHMA_643_) and poly(2-vinylpyridine)-*b*-poly(tert-butyl methacrylate)-*b*-poly(cyclohexyl methacrylate) (P2VP_25_-*b*-P*t*BMA_12_-*b*-PCHMA_173_) di- and triblock copolymers, each of a number average molecular weight *M_n_* of 14,100 g/mol, 108,000 g/mol and 33,300 g/mol, respectively, were synthesized by living anionic polymerization in THF in the presence of n-Butyllithium (n-BuLi) at −75 °C and through the employment of well-established procedures [57,58,59], and further used in this work. Their chemical structures are presented in Figure 2.

The reagents used for the preparation of copolymer solutions were toluene (C_6_H_5_CH_3_, 98%) and 1,4-dioxane (C_4_H_8_O_2_, 99.5%), both purchased from the chemical company (Iasi, Romania). Copolymer solutions were generated by dissolving 10 mg of copolymer powder in 1 mL of solvent, followed by gentle stirring. To favor the dissolution of the copolymers in the solvent, the polymeric solutions were additionally subjected to annealing at a temperature of 70 °C in a silicon oil bath (ONE 7-45, Schwabach, Germany) for 30 min.

Thin copolymer films with thicknesses of ~97 nm (P2VP_37_-*b*-PB_188_), ~103 nm (P2VP_181_-*b*-PCHMA_643_) and ~99 nm (P2VP_25_-*b*-P*t*BMA_12_-*b*-PCHMA_173_), determined by the atomic force microscopy (AFM), were obtained by spin casting the copolymer solutions onto silicon wafers (using a WS-650mz23nppb spin-coater from Laurell Technologies Corporation, North Wales, PA, USA) at a deposition speed of 2000 rpm for 30 s. The film thickness was determined by scratching the film and then measuring the profile depth of the corresponding scratch using the AFM technique. Silicon wafers of type 4PO/5-10/380±15/SSP/TTV<5 were purchased from Siegert Wafer GmbH (Aachen, Germany) and were subjected to UV-ozone treatment for 20 min (in a PSD Pro Series-Digital UV Ozone System from Novascan; Boone, IA, USA) prior to their further use.

For the rich swelling and corresponding deswelling of the BCP films through their exposure to solvent vapor in a quasi-confined environment, a homemade C-SVA setup consisting of an aluminum sample chamber of a depth of less than 1 mm was used. The bottom of the sample chamber was thermally separated from the rest of the chamber and further connected to a high-performance Peltier element (15.4 V/8.5 A from Stonecold) (see the schematics in Figure 1). The 100 W powered Peltier module can display a maximum temperature difference ΔT of ~58 °C between the two sides in appropriate circumstances. The temperature of the Peltier module (i.e., the temperature of the bottom of the chamber and thus, the sample temperature) can be precisely regulated via a temperature controller (TCM U 10A from Electron Dynamics Ltd.; Southampton, UK) that receives feedback from a PT100 temperature sensor located in the chamber in the vicinity of the sample. The PT100 sensor continuously communicates the sample temperature to the controller. The latter is driven by a 12 V/10 A power supply and changes the strength and direction of the electric current depending on whether it needs to heat or to cool the system. At the same time, on the other side of the Peltier module there is an aluminum heat sink and a fan that help equalize the ΔT temperature. Moreover, the temperature controller is commanded by a computer software that uses proportional integral derivative technology to accurately set the temperature within the desired time. Therefore, with this homemade equipment, it is possible not only to control the temperature of the sample with a precision of 0.01 °C, but also to maintain a constant temperature over time. Furthermore, the time required to reach the desired temperature can vary between a few seconds and several hours. This means it is possible to precisely tune the rate at which the sample temperature changes and thus avoid even weak variations in temperature, which generally appear when reaching a specific temperature setpoint. This is especially important when the amount of solvent vapor condensed on the sample during rich-swelling of BCP films, must be maintained constant for a specific time. Furthermore, the sample chamber is saturated with solvent vapors using a nitrogen-based “bubbling” system connected to a flow meter. The latter allows the number of solvent vapors introduced in the chamber to be precisely regulated.

The following experimental procedure was used to swell-rich thin BCP films in a quasi-confined environment saturated with solvent vapors (note that this is not a universal sample processing recipe). Firstly, the BCP film was enclosed in the sample chamber. Then, while the chamber was heated up to 40 °C, a desired quantity of solvent vapor was being introduced inside. Next, the film temperature was set to 15 °C. While the temperature was decreasing, with a rate of 0.3 °C/s, at around 22–25 °C (the real temperature value of this range always depends on the amount, temperature, and type of the solvent vapors being “bubbled” into the chamber) the solvent vapors started to condense gradually on the surface of the BCP film. The latter started to swell and, consequently, a change in the interference colors could be observed under the optical microscope. This change in the interference colors was associated with the film thickness and further used to continuously determine the thickness of the film in its swollen state (note that an interference colors-film thickness calibration was generated with the help of the AFM technique before the start of the swelling experiments; see additional details on the procedure elsewhere [36,55,60]). At 15 °C there should be enough solvent vapors condensed on the film to transform it into a quasi 2D “solution” with a polymer concentration (*c_p_*) of about several percents (this concentration was determined as a ratio between the initial film thickness and the thickness of the swollen film; see further details here [36,55,60]). In case the change in the interference colors could not be observed at 15 °C (again, this is not a standard temperature, its value can be higher or lower depending on the initial film thickness or the desired degree of film swelling, etc.), it was necessary to further lower the sample temperature to around 12–14 °C in order to favor even more the condensation of solvent vapors on the BCP film surface and thus, to swell the film more. After about one minute at several percents low *c_p_*, we have reversed the process and the sample temperature was increased very slowly back to 40 °C, with a rate of only 0.01 °C/s (this lower rate was used in order to allow polymer molecules to pack into ordered nanostructures). Upon this time, the solvent vapors began to gradually evaporate, and the film has slowly returned to its original thickness, but with its microstructure rearranged.

For the acquisition of the AFM images, a system from Molecular Devices and Tools for Nano Technology (NT-MDT, from Spectrum Instruments Ltd., Limerick, Ireland) mounted on an Olympus IX71 optical microscope in non-contact (tapping) mode was used. The AFM measurements were performed by employing high resolution Noncontact Golden Silicon probes from NT-MDT. Such probes possessed a tip radius of a curvature smaller than 10 nm and a tip height ranging from 14 to 16 μm. The probes were also Au-coated on the detector side cantilever. The cantilever, of a length of 125 ± 5 μm, displayed a resonance frequency in the range of 187–230 kHz and a nominal force constant ranging between 1.45 and 15.1 N/m. For softer samples, PointProbe^®^ Plus Non-Contact Soft Tapping Backside Reflex coating (PPPNCSTR) NANOSENSOR probes (from NanoAndMore GmbH, Wetzlar, Germany) with reduced and more reproducible tip radius (<7 nm) were also used. Such probes exhibited a cantilever length of 150 μm, a width of 27 μm, a thickness of 2.8 μm, a cantilever force constant of 7.4 N and a cantilever resonance frequency stretching between 75 kHz and 265 kHz. The AFM images (256 × 256 lines) were acquired using a scanning speed of about 1–2 μm/s and a setpoint ranging between 9 to 12 V. The setpoint was always adjusted to secure a very soft tapping regime.

In order to demonstrate the consistency of a specific film morphology over the whole corresponding surface, the AFM images (of magnifications ranging between 15 × 15 µm^2^ down to 0.5 × 0.5 µm^2^) were measured in at least three different regions on all samples. Indeed, with this approach we have inferred that each thin film exhibited the same type of morphologies over its whole surface. Note that we have not measured the films right near their edges, as in such regions inhomogeneities in film thickness and other fabrication defects associated with the film spin casting procedure could be often observed.

## 3. Results and Discussions

**Figure 3:**
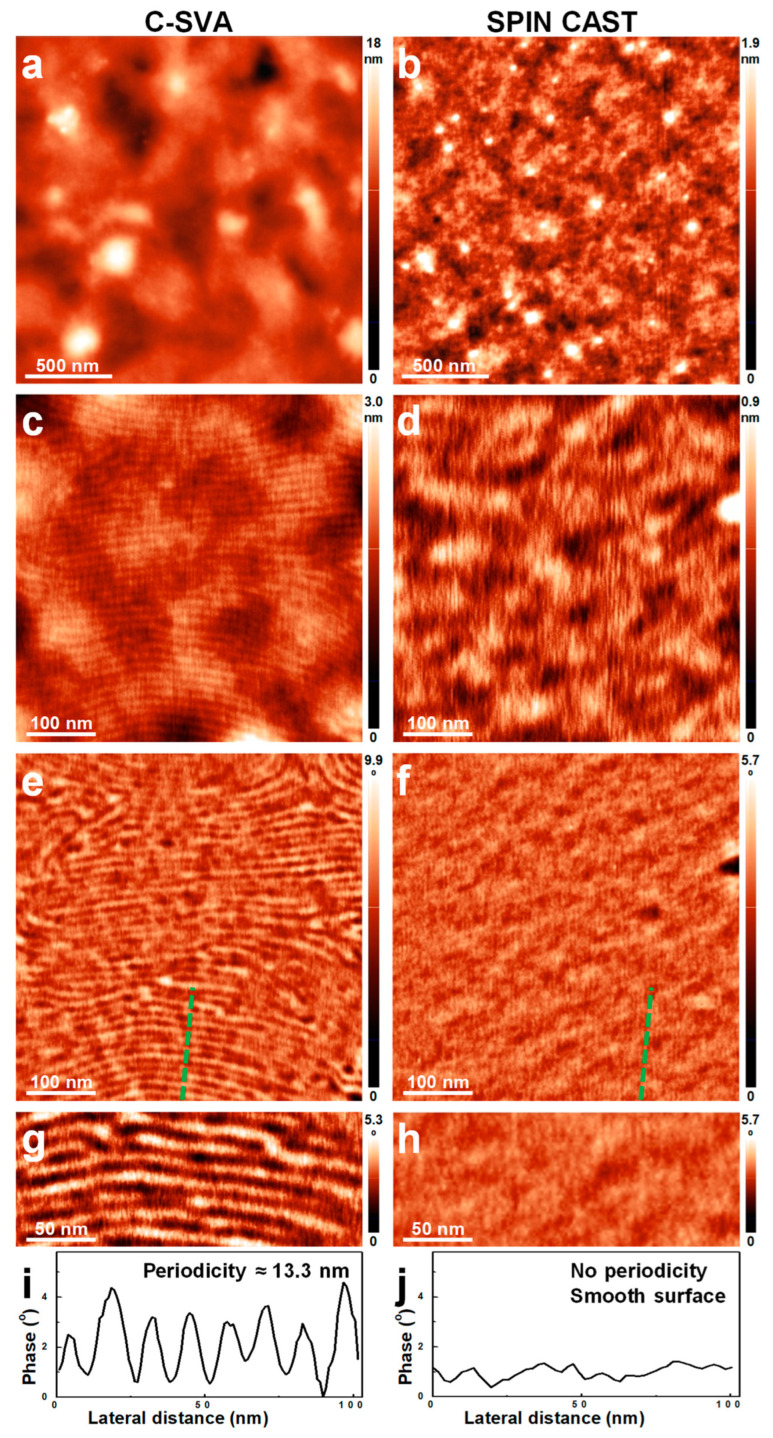
AFM height (**a**–**d**) and phase (**e**–**h**) micrographs depicting the morphology observed on the surface of a thin film of P2VP_37_-*b*-PB_188_ after (**a**,**c**,**e**,**g**) and before (**b**,**d**,**f**,**h**) its exposure to toluene vapors. While the height (**c**) and phase (**e**) micrographs represent each a magnification of a certain region from (**a**), the micrographs in (**d**) and (**f**) correspond each to a zoom-in of (**b**). Moreover, the micrographs presented in (**g**) and (**h**) are each a zoom-in of the images shown in (**e**) and (**f**), respectively. (**i**,**j**) Profile cross-sections corresponding to the dotted lines indicated in (**e**) and (**f**) and emphasizing the lateral dimensions measured after (**i**) and before (**j**) the exposure of the BCP film to toluene vapors.

Figure 3 shows a comparison of the AFM images depicting both a thin film of P2VP_37_-*b*-PB_188_ diblock copolymer that was swollen-rich via its consistent exposure to solvent vapors in a quasi-confined environment, and its unprocessed (i.e., simply as spin cast) counterpart film. The results have demonstrated that while the unprocessed P2VP_37_-*b*-PB_188_ film exhibited a featureless, yet smooth surface topography (surface roughness ~0.1 nm; Figure 3b,d), the film processed using the C-SVA method displayed, on its surface, periodic parallel stripe domains with a roughness bellow 0.3 nm (Figure 3a,c). Furthermore, when comparing the phase images shown in Figure 3e–h, it became clear that only the C-SVA-processed film displayed highly ordered stripe domains. Moreover, the periodicity of stripes, as measured by the AFM technique, was about 13.3 nm (Figure 3g,i). In contrast, the corresponding unprocessed film displayed random structures with no shape specificity (Figure 3h,j). Additionally, as it could be observed in Figure 3g, the parallel stripes consisted of alternating darker and lighter domains of an average width slightly lower than 7 nm. This value was extracted by combining many cross-sectional measurements performed on different regions of the sample. Most probably, the darker domains corresponded to the softer, yet rubbery PB block [61] (exhibiting a glass transition temperature *T_g_* smaller than −90 °C [62]), while the lighter domains represented the stiffer P2VP block (note that, as a polyelectrolyte [63], it is expected that the P2VP block would adopt a more rigid rod-like conformation due to the chain repulsion in the “film-solution”; *T_g_*~104 °C [64]). By considering that (i) the dimension of a vinyl-pyridine monomer unit is about 0.25 nm [65,66], (ii) the P2VP block tends to fully extend in solutions [67] and (iii) according to the previous studies, ordered structures formed once the BCP films were transformed (through the rich swelling in solvent vapors) into quasi 2D “solutions” containing only several percents of polymer [36,55], the P2VP domains comprised of fully extended length of the P2VP block, containing 37 monomer units, would be expected to overreach 9 nm. This maximal value is somewhat higher than the value of less than 7 nm determined from the AFM measurements for the width of the brighter P2VP domains and could indicate that the P2VP chains were not completely stretched within the observed lamellar morphology. This is highly expected, as toluene is not a good solvent for the P2VP block [68,69] and it might limit the chain mobility (and thus, the degree of chain stretching) through the toluene-P2VP interaction [69]. Instead, toluene is a frequently used solvent for the PB block [70,71]. With the PB-toluene interaction parameter χ varying between about 0.38 and 0.45 (this range was inferred from the studies performed of ternary polystyrene-polybutadiene systems in toluene; the interaction parameter was shown to be dependent on both the weight fraction of toluene and the molecular weight of the PB block [71]), toluene is therefore able to efficiently swell this rubbery block [70] and to further facilitate an eventual phase separation from the P2VP block.

Nonetheless, to the best of our knowledge, the aforementioned lamellar morphology was not reported before for any BCP systems made of P2VP and PB blocks. Although studies available in the literature reported on the development of a variety of microphase-separated structures such as spherical, cylindrical, and wormlike micelles, as well as vesicles [67], ordered lamellar morphologies and structures experiencing transitions to ribbons were only reported for other triblock and diblock copolymers containing P2VP, such as PS-*b*-P2VP, P4VP-*b*-P2VP, poly(methyl methacrylate)-*b*-poly(2-vinylpyridine) (PMMA-*b*-P2VP) or poly(hexyl isocyanate)-based P2VP-*b*-PHIC-*b*-P2VP [72,73,74,75,76,77]. Interestingly, the width of P2VP_37_-*b*-PB_188_ lamellar domains measured in this study (i.e., 13.3 nm) is smaller as compared to the width of P4VP_34_-*b*-PB_207_ lamellar domains reported recently to be around 23 nm [54], although the estimated length of the fully extended P4VP block is rather comparable. This could be caused by the shorter nature of the PB block within the P2VP_37_-*b*-PB_188_ BCP system, or/and by the possibility of the shorter soft blocks to adopt significantly more compact spatial arrangements.

**Figure 4:**
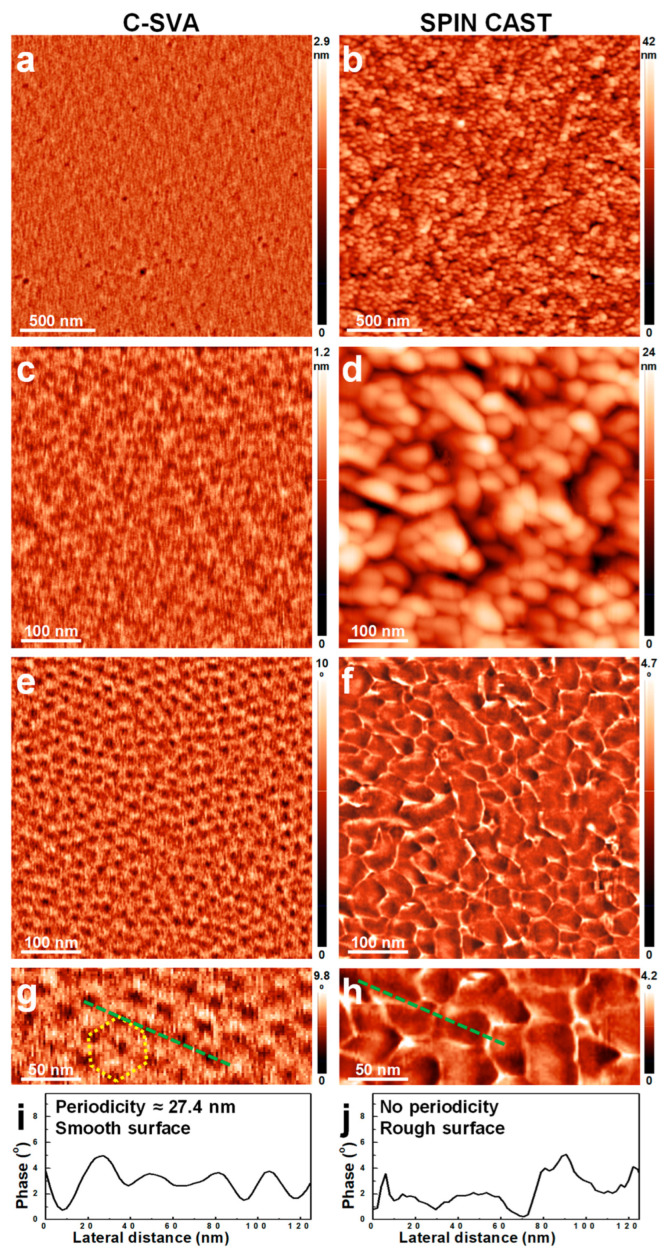
AFM height (**a**–**d**) and phase (**e**–**h**) micrographs depicting the morphology observed on the surface of a P2VP_181_-*b*-PCHMA_643_ film after (**a**,**c**,**e**,**g**) and before (**b**,**d**,**f**,**h**) its rich swelling (and subsequent deswelling) in 1,4-dioxane vapors. While the height (**c**) and phase (**e**) micrographs represent each a zoom-in of a region depicted in (**a**), the micrographs in (**d**) and (**f**) are corresponding to a zoom-in of a region portrayed in (**b**). Moreover, the micrographs presented in (**g**) and (**h**) are each a zoom-in of the images shown in (**e**) and (**f**), respectively. (**i**,**j**) Profile cross-sections corresponding to the dotted lines indicated in (**g**) and (**h**) and emphasizing the lateral dimensions measured after (**i**) and before (**j**) the exposure of the BCP film to 1,4-dioxane vapors. The purpose of the dotted shape in (**g**) is for the eye guiding only.

When the PB block was replaced with a PCHMA block, the resulting P2VP_181_-*b*-PCHMA_643_ BCP system also showed, when swollen-rich, a strong tendency towards the self-assembly. In this case, the rich exposure of P2VP_181_-*b*-PCHMA_643_ films to 1,4-dioxane vapors led to a morphology comprised of hexagonally packed core-shell self-assembled micelles made of a P2VP core and a PCHMA corona (Figure 4a,c,e), in agreement with the expectations generated by the previous reports on micellization in solutions available in the literature (note that due to both the nature of interactions and volume fraction of the blocks, this BCP is expected to self-assemble into micelles) [58,78,79,80]. The lateral periodicity of hexagonally packed micelles was determined to be over 27 nm (Figure 4g,i). In comparison, the unprocessed P2VP_181_-*b*-PCHMA_643_ molecules formed tens of nanometers large structures of, most probably, poorer molecular arrangements (Figure 4b,d,f). These structures often displayed a quasi-circular shape, with some displaying also more elongated shapes (Figure 4d,f). All structures were randomly packed and no clear lateral periodicity could be detected (Figure 4h,j). Moreover, this unprocessed BCP film exhibited a much larger surface roughness as compared to its counterpart film processed using the C-SVA method (2.8 nm vs. 0.1 nm). To the best of our knowledge, the hexagonal packing of P2VP_181_-*b*-PCHMA_643_ micelles on solid surfaces was not reported until now. The only available reports are discussing some disordered micellar films of P2VP-*b*-PCHMA [80], eventually loaded with metallic nanoparticles [81], and other micellar films made of more or less similar PS-*b*-P2VP [74,77,82], polyisoprene-*b*-poly(2-vinylpyridine) (PI-*b*-P2VP) [83], or poly(cyclohexyl methacrylate-*b*-(diethoxyphosphoryl)methyl methacrylate) (PCHMA-*b*-PDEPMMA) [84]. Films covered with spherical structures made of poly(dimethylsiloxane)-*b*-poly(2-vinylpyridine) (PDMS-*b*-P2VP) were also reported in the literature [85].

**Figure 5:**
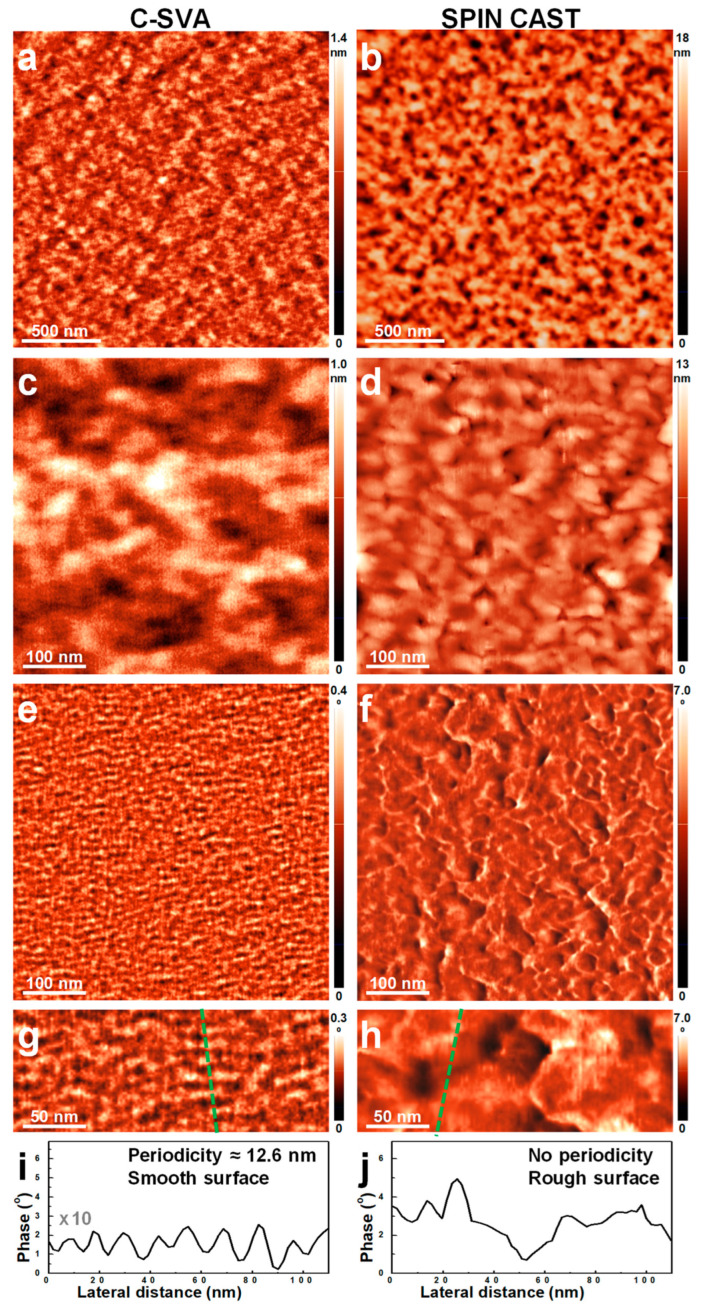
AFM height (**a**–**d**) and phase (**e**–**h**) micrographs depicting the morphology observed on the surface of a thin film of P2VP_25_-*b*-P*t*BMA_12_-*b*-PCHMA_173_ after (**a**,**c**,**e**,**g**) and before (**b**,**d**,**f**,**h**) its exposure to 1,4-dioxane vapors in a rather confined sample chamber. While the height (**c**) and phase (**e**) micrographs represent each a zoom-in of a region depicted in (**a**), the micrographs in (**d**) and (**f**) are corresponding to a zoom-in of a region portrayed in (**b**). Moreover, the micrographs presented in (**g**) and (**h**) are each a zoom-in of the images shown in (**e**) and (**f**), respectively. (**i**,**j**) Profile cross-sections corresponding to the dotted lines indicated in (**g**) and (**h**) and emphasizing the lateral dimensions measured after (**i**) and before (**j**) the exposure of the BCP film to 1,4-dioxane vapors.

Upon a significant reduction of the P2VP and PCHMA blocks from 181 to 25 monomers and from 643 to 173 monomers, respectively, while adding in between them another short block of 12 P*t*BMA monomer units, the resulting triblock copolymer films have shown more peculiar surface morphologies (Figure 5). More exactly, the P2VP_25_-*b*-P*t*BMA_12_-*b*-PCHMA_173_ film that was swollen-rich using the C-SVA method led to a smooth surface (roughness < 0.1 nm) covered with tiny spherical structures (Figure 5a,c). Repeated measurements on more than 25 such structures observed in Figure 5e revealed that their average diameter was about 12.5±0.8 nm. Because of the volume fraction displayed by its constituent blocks, this triblock copolymer system is known to form micelles in solutions. Such micelles were shown to exhibit a hydrodynamic radius of 17.9 nm [78]. Therefore, we have inferred that the spherical structures observed in Figure 5e were P2VP_25_-*b*-P*t*BMA_12_-*b*-PCHMA_173_ micelles. Moreover, the lower diameter of these micelles (i.e., 12.5±0.8 nm) that has been determined from the AFM measurements in dry films, indicates that the micelles formed in quasi-2D “solutions” (i.e, in rich-swollen films) supposedly underwent further conformational changes (i.e., shrinking) upon the evaporation of the solvent and subsequent drying of the films. Furthermore, even though the micelles seemed to be randomly distributed on the surface (Figure 5e), a more detailed analysis revealed that oftentimes, there were regions on the surface where these micelles had the tendency to pack into parallel stripe-like periodic superstructures (Figure 5g). The latter displayed a lateral periodicity matching well to the diameter of the micelles (Figure 5i). Thus, the P2VP_25_-*b*-P*t*BMA_12_-*b*-PCHMA_173_ triblock copolymer system led, upon its controlled exposure to 1,4-dioxane vapors, to a mixed morphology comprised of micelles that were both randomly oriented on the surface and often packed in ordered stripe-like superstructures. Instead, the spin cast P2VP_25_-*b*-P*t*BMA_12_-*b*-PCHMA_173_ film that was not exposed to solvent vapors showed a much rougher (roughness~1 nm), “porous”-like surface morphology (Figure 5b), comprised of 50–100 nm large structures. Such structures were characterized by “quasi-circular” or elongated shapes (Figure 5d,f). No clear periodicity could be detected on such unexposed samples (Figure 5j).

## 4. Conclusions

By utilizing an optimized polymer processing approach based on solvent vapor annealing in a space-confined environment, we have succeeded, under well-controlled temperature and polymer concentration conditions, to swell-rich various block copolymer thin films and to generate previously unreported self-assembled surfaces comprised of periodic lamellar and hexagonal nanostructures, respectively. These studies represent a step forward towards engineering prospective antimicrobial surfaces comprised of idealized nanostructures self-assembled from BCPs containing quaternized P2VP and P4VP blocks.

## Data Availability

The data presented in this study are available on request from the corresponding author.
